# Whole-Genome Sequence of Rhizobacterium *Sphingomonas* sp. Strain 7/4-4, Isolated from the Root Nodule of Astragalus tugarinovii Basil Growing in the Russian Arctic

**DOI:** 10.1128/mra.00141-23

**Published:** 2023-05-15

**Authors:** Polina Guro, Denis Karlov, Irina Kuznetsova, Anna Sazanova, Irina Alekhina, Andrey Belimov, Vera Safronova

**Affiliations:** a All-Russia Research Institute for Agricultural Microbiology, St. Petersburg, Russian Federation; b Arctic and Antarctic Research Institute, St. Petersburg, Russian Federation; Loyola University Chicago

## Abstract

The complete genome sequence of *Sphingomonas* sp. strain 7/4-4, which was isolated from the root nodule of the circumpolar legume Astragalus tugarinovii Basil, is reported. The assembly contains 4,423,370 bp in 1 circular chromosome, with a GC content of 65.94%. The genome sequence of strain 7/4-4 could provide insights into the metabolic potential of Arctic rhizobacteria.

## ANNOUNCEMENT

The Lena River delta region has a dry continental Arctic climate characterized by very low temperatures, low precipitation levels, and wet polygonal tundra with a short vegetation period (1). Because rhizosphere microorganisms are an integral part of the ecosystem, they are natural partners that can modulate local and systemic mechanisms in plants to provide protection under adverse external conditions. Strain 7/4-4 was isolated from the nodule of Astragalus tugarinovii Basil, which was collected near the Lena River delta, from the southern slope of Lake Sevastian-Kuele (71.514919°N, 128.80375°E), Sakha Republic, Russia. Individual nodules were sterilized for 1 min in 70% ethanol, rinsed, homogenized in sterile tap water, and plated on a yeast extract-maltose-sucrose agar (YMSA) medium. Colonies of strain 7/4-4 appeared after 5 days of incubation at 28°C. The strain was purified by repeated restreaking on the medium. DNA was isolated without size selection using the DNeasy blood and tissue kit (Qiagen, Germany), according to the manufacturer's recommendations, from a pure culture grown in Reasoner’s 2A (R2A) broth at 28°C for 48 h. The library was prepared with the SQK-LSK109 ligation sequencing kit and the EXP-NBD104 native barcoding expansion 1–12 kit (Oxford Nanopore Technologies [ONT], Oxford, UK), skipping the DNA-shearing step, and was sequenced using a MinION sequencer with the R.9.4.1 flow cell (ONT). Base calling was performed using Guppy v5.0.11 (2). The sequencing resulted in 112,744 reads, with an average read length of 6,657 bp. Quality control of the raw reads was provided by NanoStat v1.6.0 (3). *De novo* assembly was carried out using Flye v2.9 (4) and produced 1 circular contig with ~160× coverage. The resulting chromosome was rotated to start from the *dnaA* gene using Circlator v1.5.5 (5). Default parameters were used for all software unless otherwise noted. Assembly statistics were provided by QUAST v5.0.2 (6). The total size and *N*_50_ value for the assembled genome were 4,423,370 bp, with a GC content of 65.94%. Phylogenetic analysis based on 16S rRNA BLASTn comparisons indicated that strain 7/4-4 was affiliated with the genus *Sphingomonas* and closely related to the type strains Sphingomonas psychrotolerans Cra20 (GenBank accession number CP024923.1) and Sphingomonas hengshuiensis WHSC-8 (GenBank accession number NR_145956.2), with identity levels of 97.44% and 97.92%, respectively (7). Analysis of the average nucleotide identity (ANI) with respect to strains Cra20 and WHSC-8 yielded ANIb values of 83.90% and 78.46%, respectively, which fall below the threshold for species delineation (8). We suggest that strain 7/4-4 may belong to a new *Sphingomonas* species. Assembly annotation using PGAP v6.4 resulted in 3,786 protein-coding genes and 59 RNA genes (6 rRNA genes, 50 tRNA genes, and 3 noncoding RNA genes) (9). The genome of *Sphingomonas* sp. strain 7/4-4 contains multiple putative genes of stress response subsystems and resistance to toxic compounds (fluoroquinolones, copper, zinc, and cadmium), as well as auxin genes and a set of symbiotic genes (*nifABLJ*, *fixALHRSB*, and *nodPQVWJI*), which could act as biotic elicitors for plant defense systems and adaptation to the adverse environmental conditions.

### Data availability.

All data are available at GenBank under BioProject accession number PRJNA881974. The BioSample accession number is SAMN32362222. The raw MinION data can be found under SRA accession number SRR22971173, and the assembly accession number is GCF_027941955.1.
